# Binding of human recombinant mutant soluble ectodomain of FGFR2IIIc to c subtype of FGFRs: implications for anticancer activity

**DOI:** 10.18632/oncotarget.12067

**Published:** 2016-09-16

**Authors:** Zhong Liu, Ge Liu, Guang-Lin Zhang, Jun Li, Yan-Qing He, Shu-Shu Zhang, Yi Wang, Wei-Yi He, Guo-Hua Cheng, Xuesong Yang, Jun Xu, Ju Wang

**Affiliations:** ^1^ Institute of Biomedicine, Guangdong Provincial Key Laboratory of Bioengineering Medicine, National Engineering Research Center of Genetic Medicine, Jinan University, Guangzhou 510632, China; ^2^ Key Laboratory for Regenerative Medicine of the Ministry of Education, Division of Histology and Embryology, Medical College, Jinan University, Guangzhou 510632, China; ^3^ Research Center for Drug Discovery, School of Pharmaceutical Sciences, Sun Yat-sen University, Guangzhou 510006, China; ^4^ College of Pharmacy, Jinan University, Guangzhou 510632, China

**Keywords:** heterocomplex, FGF-2, soluble FGFR2, tumor inhibition, angiogenesis

## Abstract

FGFRs are considered essential targets for cancer therapy. We previously reported that msFGFR2c, a Ser252Trp mutant soluble ectodomain of FGFR2IIIc, inhibited tumor growth by blocking FGF signaling pathway. However, the underlying molecular mechanism is still obscure. In this study, we reported that msFGFR2c but not wild-type soluble ectodomain of FGFR2IIIc (wsFGFR2c) could selectively bind to c subtype of FGFRs in the presence of FGF-2. Thermodynamic analysis demonstrated that msFGFR2c bound to wsFGFR2c in the presence of FGF-2 with a K value of 6.61 × 10^5^ M^−1^. Molecular dynamics simulations revealed that the mutated residue Trp252 of msFGFR2c preferred a π-π interaction with His254 of wsFGFR2c. Concomitantly, Arg255 of msFGFR2c and Glu250 of wsFGFR2c adjusted their conformations and formed three H-bonds. These two interactions therefore stabilized the final structure of wsFGFR2c and msFGFR2c heterocomplex. In FGFR2IIIc-positive/high FGF-2-secreted BT-549 cells, msFGFR2c significantly inhibited the proliferation and induced apoptosis by the blockage of FGF-2-activated FGFRs phosphorylation, also the growth and angiogenesis of its xenograft tumors implanted in chick embryo chorioallantoic membrane model. While weaker the above inhibitory effects of msFGFR2c were observed on FGFR2IIIc-negative/low FGF-2-secreted MCF-7 and MDA-MB-231 cell lines *in vitro* and *in vivo*. Moreover, msFGFR2c significantly inhibited the proliferation of FGFR1IIIc-positive NCI-H1299 lung cancer cells by the suppression of FGF-2-induced FGFR1 activation and suppressed the growth of NCI-H1299 transplanted tumors in nude mice. In sum, msFGFR2c is a potential anti-tumor agent targeting FGFR2c/FGFR1c-positive tumor cells. These findings also provide a molecular basis for msFGFR2c to disrupt the activation of FGF signaling.

## INTRODUCTION

It is well known that receptor tyrosine kinases (RTKs) play important roles in the development and progression of human cancers [1]. Several strategies to block RTK signal pathways have been developed, such as neutralizing antibodies targeting ligands or receptors, small molecular RTK inhibitors (TKIs) and so on [2, 3]. However, the most severe perplexity to utilize specific-antibodies or TKIs in clinic is that tumor cells can often survive through activating redundant RTK pathways. Nowadays, the development of soluble receptors is an emerging treatment strategy to effectively block RTK signals [4–10]. Soluble receptors are the soluble extracellular domains of the membrane receptors such as soluble epidermal growth factor receptor (sEGFR), soluble vascular epidermal growth factor receptor (sVEGFR) and soluble fibroblast growth factor receptor (sFGFR), etc [11–13]. Generally, it is believed that soluble receptors block RTK signals through ligand trapping, i.e. lowering the effective concentrations of free ligands (FGFs, VEGFs, EGFs) available for receptors activation [14].

Different soluble receptors can selectively bind to overlapping sets of ligands [15, 16]. For example, FGF-1 can bind to almost all kinds of FGFRs; FGF-7 can only bind to FGFR2IIIb; FGF-2 can bind to FGFR1b/FGFR1c/FGFR2c/FGFR3c [17]. Similarly, soluble FGFR2c ectodomain can bind to multiple ligands, including FGF-1, FGF-2, FGF-4, FGF-6, FGF9, FGF-17, and FGF18 [18]. Thus, soluble receptors may have some advantages as anti-tumor agents since the ability of blocking RTK signaling pathways in a network type by trapping kinds of ligands.

Since recent studies showed that gene amplification, abnormal activation, or single nucleotide polymorphisms (SNPs) of FGFR2 played important roles in cancer progression [9, 19–22], FGFR2 has been recognized as a promising therapeutic target for cancers [23, 24]. Structure-function studies of FGFR2 with Ser252Trp (S252W) mutation demonstrated that the mutant receptor had altered ligand specificity and enhanced affinity for multiple FGFs compared to wild-type FGFR2 [16, 25]. Our previous study demonstrated that msFGFR2c, a soluble ectodomain of FGFR2IIIc with S252W mutation which only includes II and III Ig-like domain, inhibited tumor growth and metastasis through suppressing growth of cancer cells, enhancing apoptosis of cancer cells, and reducing tumor angiogenesis [26]. We further observed that the inhibitory effects of msFGFR2c was not only associated with the concentrations of ligand (FGF-2) but also with the endogenous expression of FGFRs in the different types of cancer cells. However, the molecular mechanism to block FGF signal is still obscure.

In this study, we reported that msFGFR2c but not wsFGFR2c (wild-type ectodomain of FGFR2c) could selectively bind to c subtype of FGFRs in the presence of FGF-2. The effects of msFGFR2c on cancer cells expressing different types of FGFRs were evaluated *in vitro* and *in vivo*. The results will be definitely a peek into the future application of msFGFR2c in cancer targeting therapy.

## RESULTS

### FGF-2-induced formation of a receptor heterocomplex consisting of msFGFR2c, membrane-bound FGFRs and FGF-2

The full length FGFRs (*FGFR1IIIb-HA*, *FGFR1IIIc-HA*, *FGFR2IIIb-HA* or *FGFR2IIIc-HA*) were transiently transfected into HEK 293T cells, and the interaction between FGFRs-HA and FITC-sFGFR2c were detected by co-immunoprecipitation and confocal microscopy analysis.

**Figure 1:**
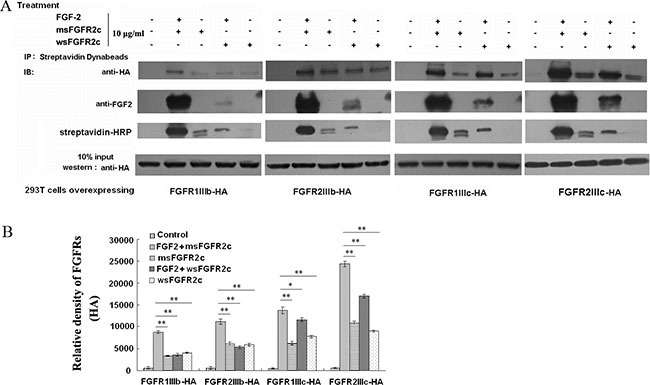
Analysis of the interaction between sFGFR2c and membrane-bound FGFRs by co-immunoprecipitation (**A**) HEK 293T cells were transfected with pcDNA3.1 *FGFR2IIIc-HA*/*FGFR2IIIb-HA*/*FGFR1IIIc-HA*/*FGFR1IIIb-HA* and treated with 10 μg/ml biotin-labeled msFGFR2c or wsFGFR2c in the presence or absence of FGF-2 for 2 h. After incubation with 50 μl of Dynabeads (R) M-270 Streptavidin at room temperature for 15 min, the beads were washed, harvested and analyzed by western blot analysis. (**B**) Quantification of expression levels of precipitated FGFR2IIIc-HA, FGFR2IIIb-HA, FGFR1IIIc-HA, and FGFR1IIIb-HA. The amounts of protein expression levels were quantified by Quantity One basic 4.6.3 software (Bio-Rad Laboratories, CA, USA). *p < 0.05; **p < 0.01.

Co-immunoprecipitation analysis showed that FGF-2 was required for the bindings of sFGFR2c to the FGFRs (Figure 1A). sFGFR2c bound to all 4 types of FGFR isoforms in the presence of FGF-2. The order of the binding amounts for sFGFR2c was FGFR2IIIc-HA>FGFR1IIIc-HA>FGFR2IIIb-HA>FGFR1IIIb-HA (Figure 1B). While in the absence of FGF-2, the amounts of msFGFR2c bound to FGFR2IIIc, FGFR1IIIc, FGFR2IIIb and FGFR1IIIb decreased dramatically as compared to FGF-2-treated group. It was observed in Figure 1A that the binding amounts of msFGFR2c to FGFRs were more than that of wsFGFR2c either in the presence or absence of FGF-2.

**Figure 2:**
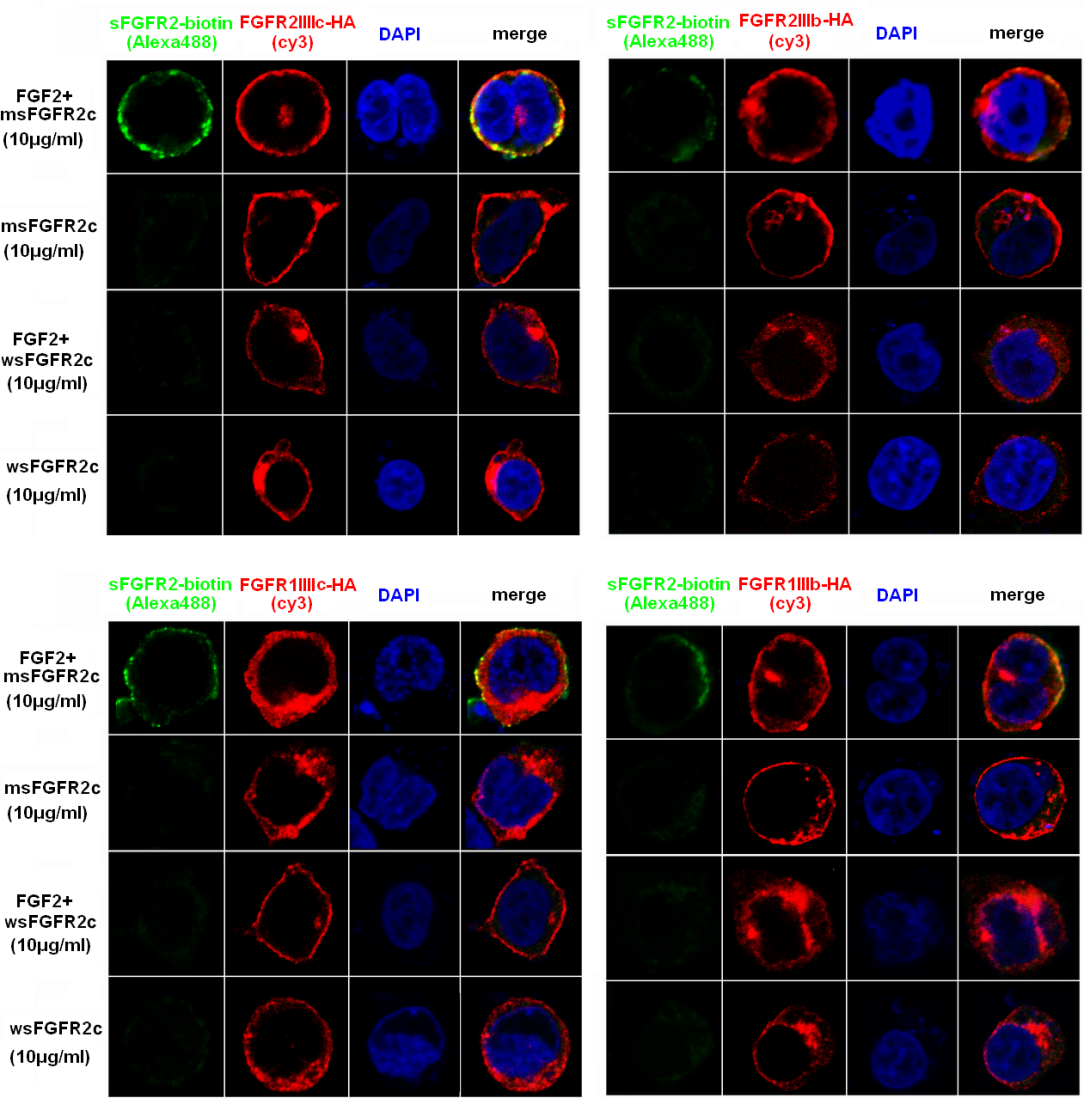
Confocal microscopy analysis of the interaction between sFGFR2c and membrane-bound FGFRs HEK 293T cells were transfected with pcDNA3.1 *FGFR2IIIc-HA* (upper left)/*FGFR2IIIb-HA* (upper right)/*FGFR1IIIc-HA* (lower left)/*FGFR1IIIb-HA* (lower right) and incubated with biotin-labeled msFGFR2c or wsFGFR2c in the presence or absence of FGF-2. Green, biotin-labeled sFGFR2c (Alexa Fluor 488); red, HA (cy3); blue pixels, DNA stained with DAPI.

Confocal microscopy analysis also showed that msFGFR2c rather than wsFGFR2c co-localized with FGFRs in the presence of FGF-2 (Figure 2), which was consistent with the co-immunoprecipitation analysis.

### Binding of sFGFR2c to FGFR2IIIc-HA in FGFR2IIIc-HA-overexpressed HEK 293T cells

**Figure 3:**
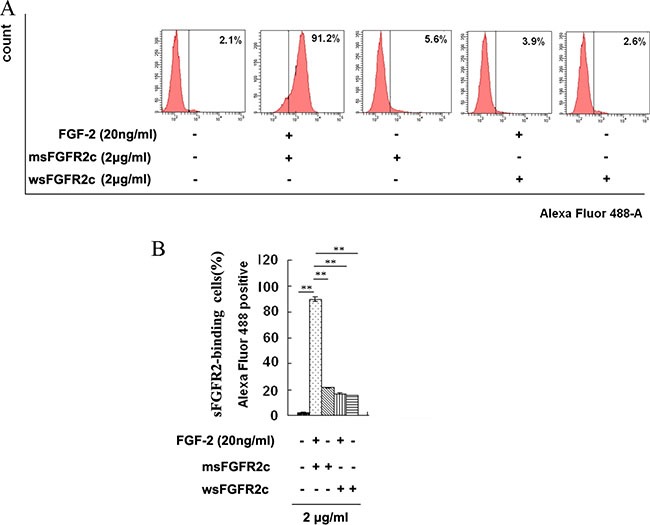
Identification of sFGFR2c binding to surface of FGFR2IIIc by flow cytometry (**A**) HEK 293T cells were transfected with pcDNA3.1 *FGFR2IIIc-HA* and incubated with biotin-labeled sFGFR2c (2 μg/ml, Alexa Fluor 488) in the presence or absence of FGF-2. The sFGFR2-binding cells were analyzed by flow cytometry. (**B**) The percentage of sFGFR2-binding cells expressed as mean fluorescence intensity. **, *p* < 0.01.

The binding of sFGFR2c to FGFR2IIIc-HA in FGFR2IIIc-HA-overexpressed HEK 293T cells was measured by flow cytometric analysis. The results in Figure 3 showed that, in the absence of FGF-2, neither wsFGFR2c nor msFGFR2c with concentration at 2 μg/ml could bind to FGFR2IIIc-HA in FGFR2IIIc-HA-overexpressed HEK 293T cells. While in the presence of FGF-2 (20 ng/ml), msFGFR2c but not wsFGFR2c with concentration at 2 μg/ml could bind to the surface of FGFR2IIIc-HA-overexpressed HEK 293T cells.

### Thermodynamic analysis of the binding of msFGFR2c to FGFR2IIIc

**Figure 4:**
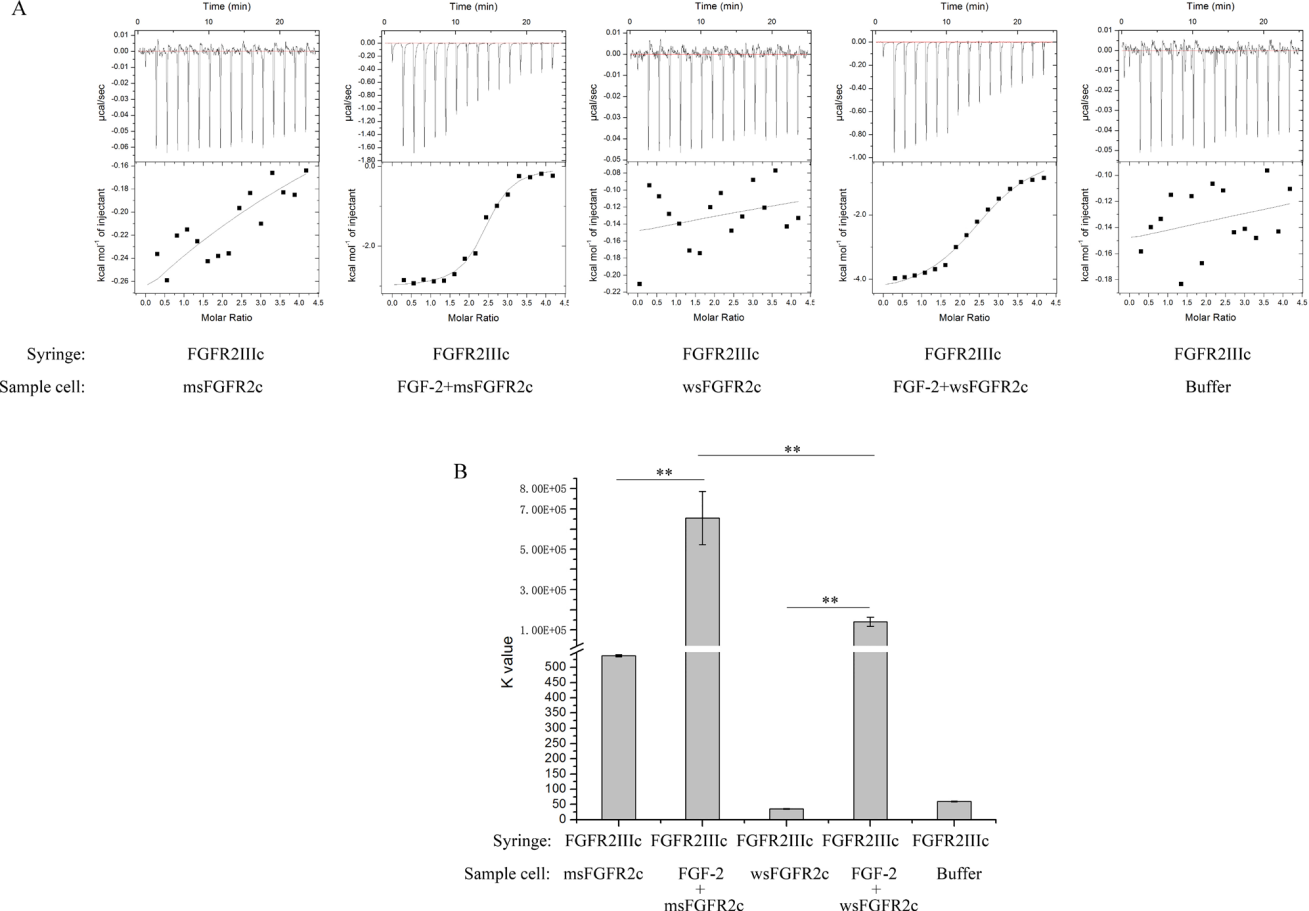
Thermodynamic analysis of the interaction between sFGFR2c and FGFR2IIIcs (**A**) Raw data (dq/dt) for injections of FGFR2IIIc into sFGFR2c in the presence or absence of FGF-2 in 25 mM HEPES containing 600 mM NaCl and 5% glycerol (pH 7.5). (**B**) The K value after peak integration and concentration normalization. **p < 0.01. The experiment was repeated three times.

To determine the binding affinity of msFGFR2c with FGFR2c, wsFGFR2c, the ectodomain of membrane FGFR2IIIc, was used to titrate with msFGFR2c in the presence or absence of FGF-2 by ITC assay. The results showed that msFGFR2c could bind to wsFGFR2c in the presence of FGF-2 with a K value of 6.61 × 10^5^ M^−1^, which was about 3-fold higher than that in wsFGFR2c-wsFGFR2c titration (Figure 4A and 4B). Nevertheless, only weak interaction between msFGFR2c and wsFGFR2c was observed in the absence of FGF-2.

### Molecular dynamics simulations of interactions between msFGFR2c and wsFGFR2c

**Figure 5:**
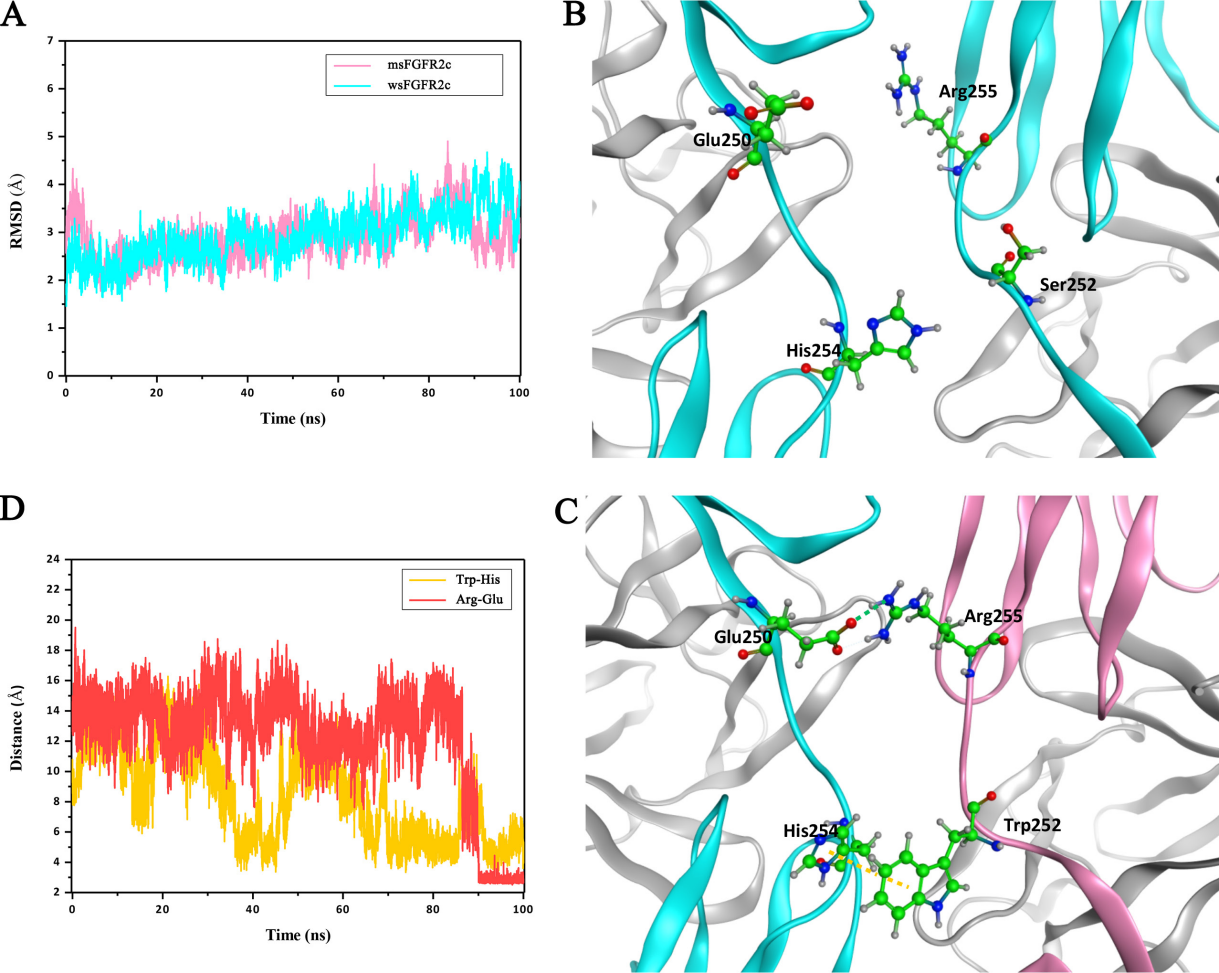
MD simulations of interactions between msFGFR2c and wsFGFR2c (**A**) RMSD for alpha carbon atoms of msFGFR2c and wsFGFR2c compared to the initial structure. (**B**) The residue Ser252 to be mutated and some other key residues of wsFGFR2c shown in stick and ball style. (**C**) Two newly formed interactions after MD simulation. The π-π interaction between residues His254 and Trp252 is shown in yellow dashed line. The hydrogen-bond interactions between residues Glu250 and Arg255 are shown in green dashed line. The msFGFR2c and wsFGFR2c chains are shown in pink and cyan ribbon models, respectively. (**D**) Distances evolution with MD simulation. The red line represents the distance between oxygen atom of the residue Glu250 and nitrogen atom of the residue Arg255. The yellow line represents the distance between two centers of mass calculated from two ring planes of the residue His254 and the mutated residue Trp252.

To explore the structural basis of interactions between msFGFR2c and wsFGFR2c, we conducted MD simulations on this heterocomplex system. As shown in Figure 5A, during 100 ns MD simulations, the whole complex got stable at around 20 ns and maintained its backbone structure in the indicated time. Although the RMSD of alpha carbon atoms presented a slight fluctuation around 80 ns than before, it reached stability during the last 10 ns. When we looked at the structures extracted from the last stable stage, a few residues showed obviously different from those in crystal structure of wsFGFR2c dimer (Figure 5B). Firstly, the mutated residue Trp252 of msFGFR2c preferred a π-π interaction with residue His254 of wsFGFR2c (Figure 5C). The distance between the centers of mass of imidazole ring in His254 and indole ring in the residue Trp252 reduced from more than 10 Å to around 4-6 Å (Figure 5D, yellow line), which first reduced at around 40 ns but raised back again soon. The second reduction happened at around 70 ns, this state remained longer than the first one, but still raised back to about 8 Å for a while. Concomitantly, the residues Arg255 of msFGFR2c and Glu250 of wsFGFR2c adjusted their conformations and formed three hydrogen bonds with distances around 2.9 Å (Figure 5C). While in wild-type wsFGFR2c dimer, the residue Ser252 was not able to contact with the residue His254, consequently the residue Arg255 did not have a comfortable position to interact with the residue Glu250 (Figure 5B).

### Endogenous expression levels of FGFRs and FGF-2 in human breast cancer cell lines BT-549, MCF-7, and MDA-MB-231

**Figure 6:**
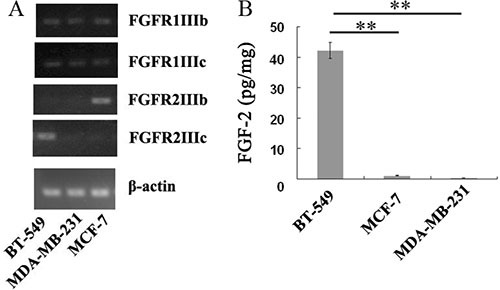
Endogenous expressions of FGFRs and FGF-2 in BT-549, MCF-7, and MDA-MB-231 cells (**A**) Analysis of mRNA expression levels of FGFR1IIIb, FGFR1IIIc, FGFR2IIIb and FGFR2IIIc in BT-549, MCF-7, and MDA-MB-231 cells by RT-PCR. (**B**) Quantitation of FGF-2 concentration in the supernatants of BT-549, MCF-7, and MDA-MB-231 cells by ELISA assays. **p < 0.01.

To understand the differences of human breast cancer cell lines in the expression levels of FGFRs and FGF-2, RT-PCR and ELISA assays were performed in three human breast cancer cell lines BT-549, MCF-7, and MDA-MB-231. As shown in Figure 6A, BT-549 and MCF-7cell lines expressing high level of FGFR2IIIc and FGFR2IIIb isoforms, respectively, whereas these two isoforms were barely expressed in MDA-MB-231 cells. In addition, Low expression levels of FGFR1IIIc and FGFR1IIIb were observed in all the three breast cancer cell lines (Figure 6A).

ELISA assays demonstrated that the concentration of FGF-2 reached 42.3 pg/mg in the BT-549 cell culture medium, whereas hardly any of FGF-2 was detected in the media of MCF-7 or MDA-MB-231 cells (Figure 6B).

### Effects of msFGFR2c on the proliferation and apoptosis of BT-549, MCF-7, MDA-MB-231 cells

**Figure 7:**
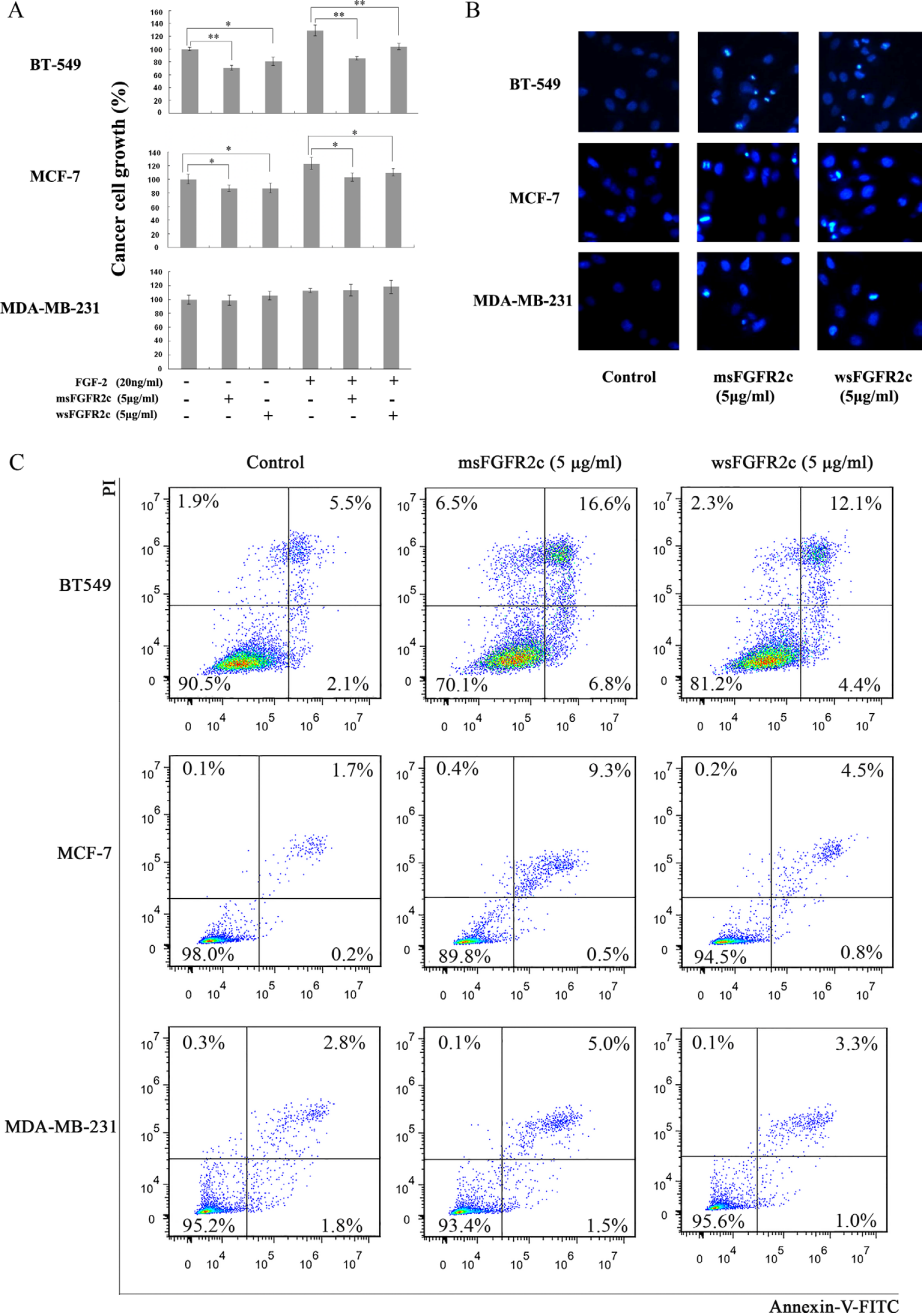
Effects of sFGFR2c on proliferation and apoptosis of BT-549, MCF-7, and MDA-MB-231 cells (**A**) Effects of sFGFR2c on the proliferation of BT-549, MCF-7, and MDA-MB-231 cells by BrdU cell proliferation assay. (**B**) Effects of sFGFR2c (5 μg/ml) on the apoptosis of BT-549, MCF-7, and MDA-MB-231 cells by DAPI staining observed under a fluorescence microscope. *p < 0.05; **p < 0.01. (**C**) Effects of sFGFR2c on the apoptosis of BT-549, MCF-7, and MDA-MB-231 cells by Annexin V-FITC/PI dual staining analysis.

The results of BrdU cell proliferation assay and DAPI staining analysis showed that msFGFR2c with concentration at 5 μg/ml significantly inhibited the proliferation and induced apoptosis in FGFR2IIIb-negative/FGFR2IIIc-positive BT-549 cells (Figure 7). In contrast, the proliferation inhibitory effects of msFGFR2c on FGFR2IIIb-positive/FGFR2IIIc-negative MCF-7 cells were weaker than on BT-549 cells and even no inhibitory effect was observed on FGFR2IIIb-negative/FGFR2IIIc-negative MDA-MB-231 cells (Figure 7A). Besides, the proliferation inhibitory effect of msFGFR2c was stronger than wsFGFR2c on BT-549 cells. The cell apoptosis analysis by Annexin V-FITC/PI dual staining in Figure 7C showed that the proportion of msFGFR2c-induced apoptosis in BT-549 cells was 23.4%. While those of in MCF-7 and MDA-MB-231 cells were 9.8% and 6.5%, respectively, which were less than in BT-549 cells, suggesting that the results of cell apoptosis analysis were consistent with that of cell proliferation assay.

### Effects of msFGFR2c on FGFR phosphorylation in breast cancer cell lines BT-549, MCF-7, and MDA-MB-231

**Figure 8:**
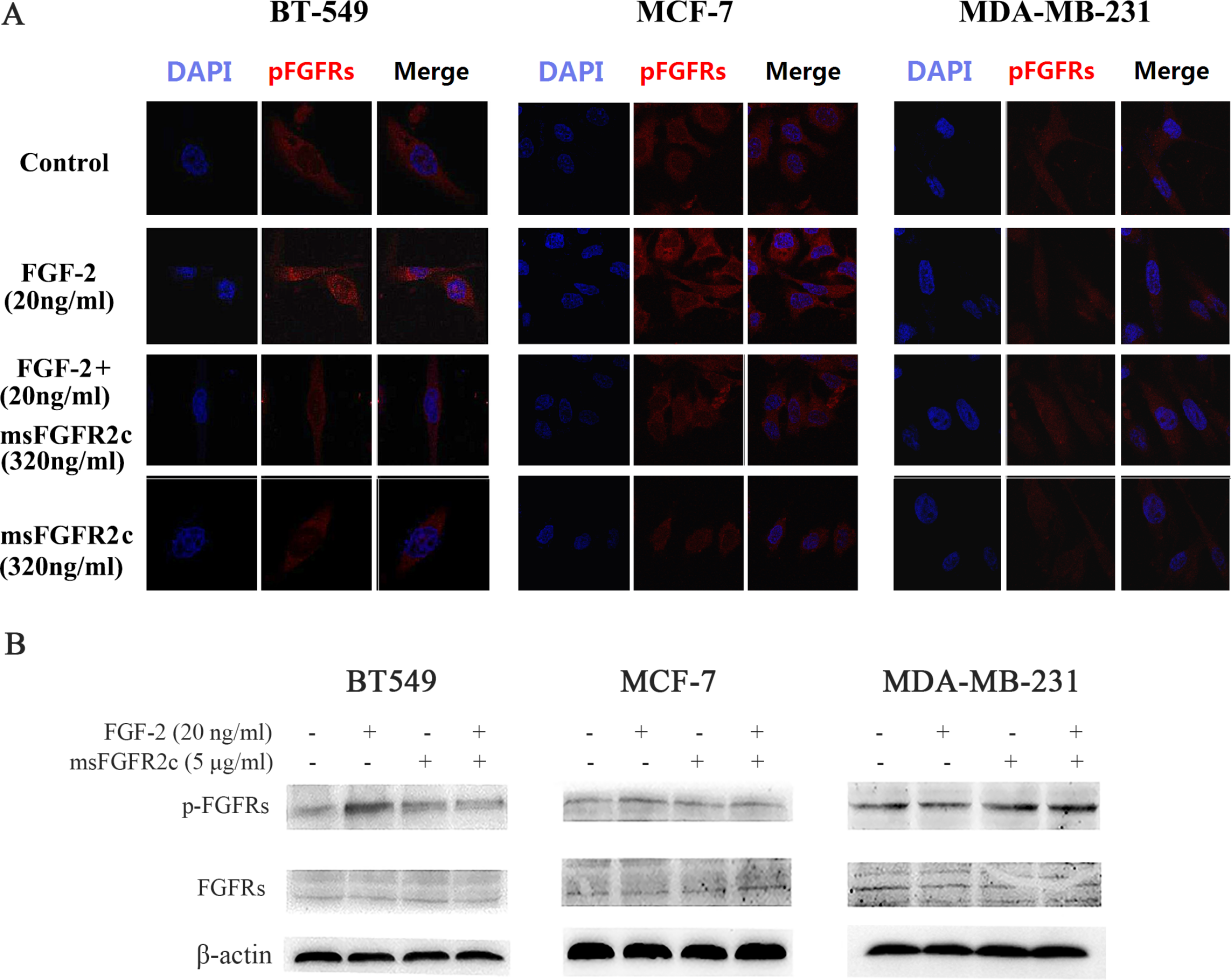
Effects of msFGFR2c on FGF-2-induced FGFR phosphorylation in BT-549, MCF-7, and MDA-MB-231 cells (**A**) msFGFR2c (320 ng/ml) was incubated with BT-549, MCF-7, and MDA-MB-231 cells, respectively, in the presence or absence of FGF-2 (20 ng/ml). The phosphorylation of FGFRs was observed by confocal microscope. (**B**) msFGFR2c (5 μg/ml) was incubated with BT-549, MCF-7, and MDA-MB-231 cells, respectively, in the presence or absence of FGF-2 (20 ng/ml). The expression levels of FGFRs and p-FGFRs were determined by western blot analysis.

Confocal microscopy analysis showed that FGF-2-activated phosphorylation level of FGFRs was almost completely blocked by msFGFR2c in BT-549 cells, and was partially inhibited in MCF-7 cells. While in MDA-MB-231 cells, FGF-2 could not activate FGFR phosphorylation (Figure 8A). Western blot analysis also confirmed the above results (Figure 8B). These results were consistent with the observations that msFGFR2c inhibited FGFR signal transduction by binding to membrane-bound FGFR2IIIc in the presence of FGF-2.

### Effects of msFGFR2c on breast cancer growth and tumor angiogenesis *in vivo*

**Figure 9:**
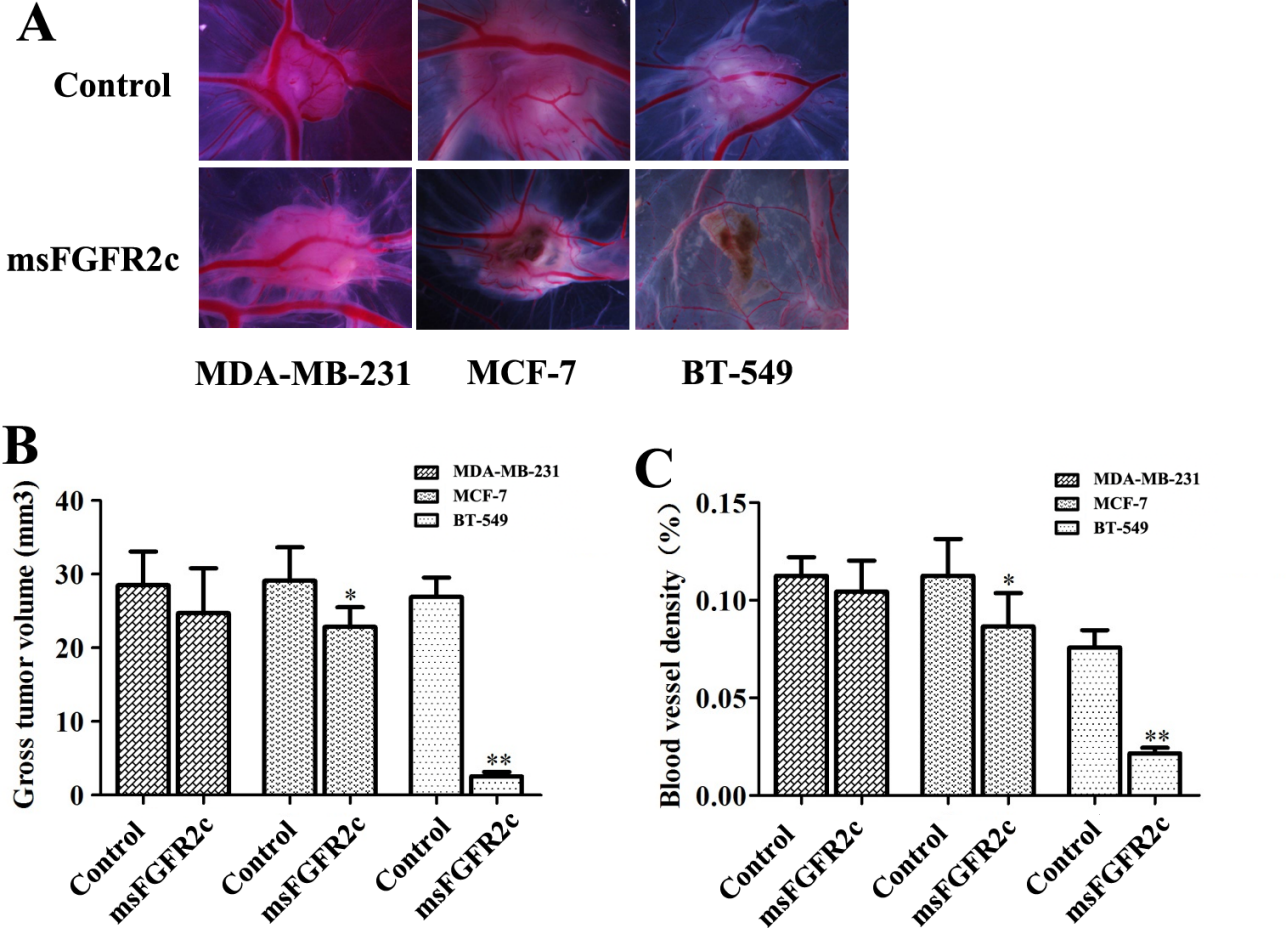
Inhibition of msFGFR2c on the growth and angiogenesis of xenograft tumors implanted by BT549, MCF-7 and MDA-MB-231 cells in CAM CAMs of fertile eggs were treated with msFGFR2c or vehicle for 3 days. (**A**) Representative images of CAMs after the indicated treatments. (**B**) Tumor volumes of msFGFR2c-treated group and vehicle in CAM (*n* = 8, *p < 0.05, **p < 0.01). (**C**) Blood vessel density expressed as percentage of vessel numbers relative to control (*n* = 8). *p < 0.05; **p < 0.01.

On the chick embryo chorioallantoic membrane (CAM), we implanted tumors of BT-549, MCF-7 and MDA-MB-231 cells. As shown in Figure 9, not only the tumor growth of BT-549 xenograft was significantly inhibited by msFGFR2c (Figure 9A), but also CAM angiogenesis was almost completely suppressed compared with the control group (Figure 9B). The inhibitory effects of msFGFR2c on tumor growth and angiogenesis of MCF-7 xenograft in CAM model were weaker than that of BT-549 xenograft, while no obvious inhibitory effect was observed on that of MDA-MB-231 xenograft.

### Effects of msFGFR2c on the proliferation and FGFR1 activation in lung cancer NCI-H1299 cells

**Figure 10:**
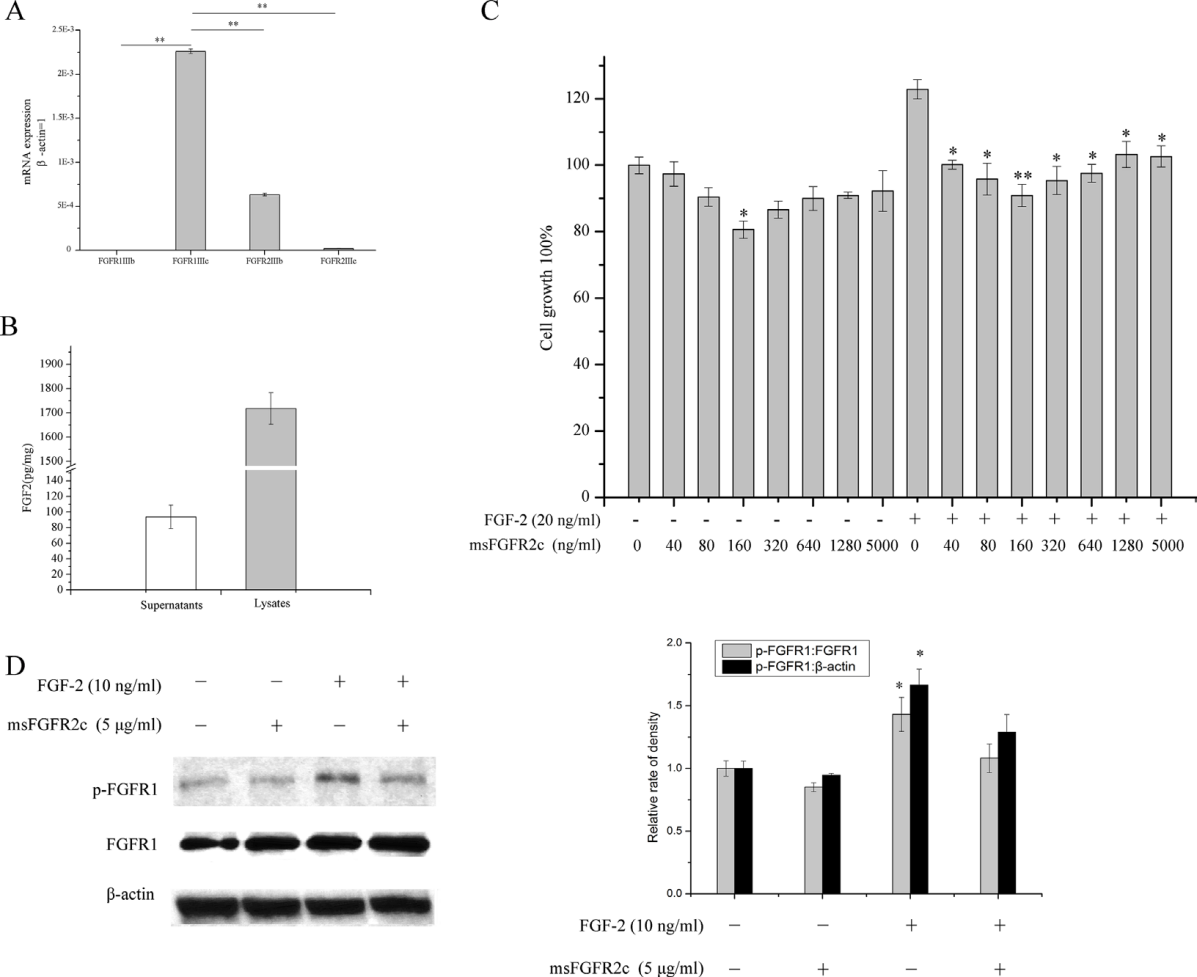
Effects of msFGFR2c on proliferation and FGFR1 phosphorylation in NCI-H1299 cells (**A**) Analysis of mRNA expression level of FGFR1IIIb, FGFR1IIIc, FGFR2IIIb and FGFR2IIIc in NCI-H1299 cells by RT-PCR analysis. (**B**) Quantitation of FGF-2 concentration in the supernatants and lysates of NCI-H1299 cells by ELISA assays. (**C**) Effects of msFGFR2c on the proliferation of NCI-H1299 cells by BrdU cell proliferation assay. (**D**) Effects of msFGFR2c on FGF-2-induced FGFR phosphorylation in the presence or absence of FGF-2 (10 ng/ml) in NCI-H1299 cells by western blot analysis (left panel). The relative rate of density of the protein expression level was quantified by Quantity One basic 4.6.3 software and presented as the percentage of control (right panel). *p < 0.05.

To extend the antineoplastic profile of msFGFR2c to different types of cancer, we further determined the effects of msFGFR2c on lung cancer growth *in vitro* and *in vivo*. As shown in Figure 10A, NCI-H1299 cell line was FGFR1IIIc and FGFR2IIIb positive since high mRNA expression level of FGFR1IIIc was observed in NCI-H1299 cells, which was about 3-fold higher than that of FGFR2IIIb. While little or hardly any FGFR1IIIb and FGFR2IIIc were detected in NCI-H1299 cells. ELISA assays demonstrated that the concentration of FGF-2 reached 93 pg/mg in the cell culture medium and 1732pg/mg in the cell lysates. (Figure 10B). BrdU cell proliferation assay in Figure 10C showed that msFGFR2c significantly inhibited the proliferation of NCI-H1299 cells in the presence of FGF-2. Then we determined the expression levels of FGFR1 and phosphorylated FGFR1 after the treatment of msFGFR2c. The results showed that msFGFR2c significantly suppressed FGF-2-activated phosphorylation level of FGFR1 in NCI-H1299 cells (Figure 10D).

### Effects of msFGFR2c on lung cancer growth *in vivo*

**Figure 11:**
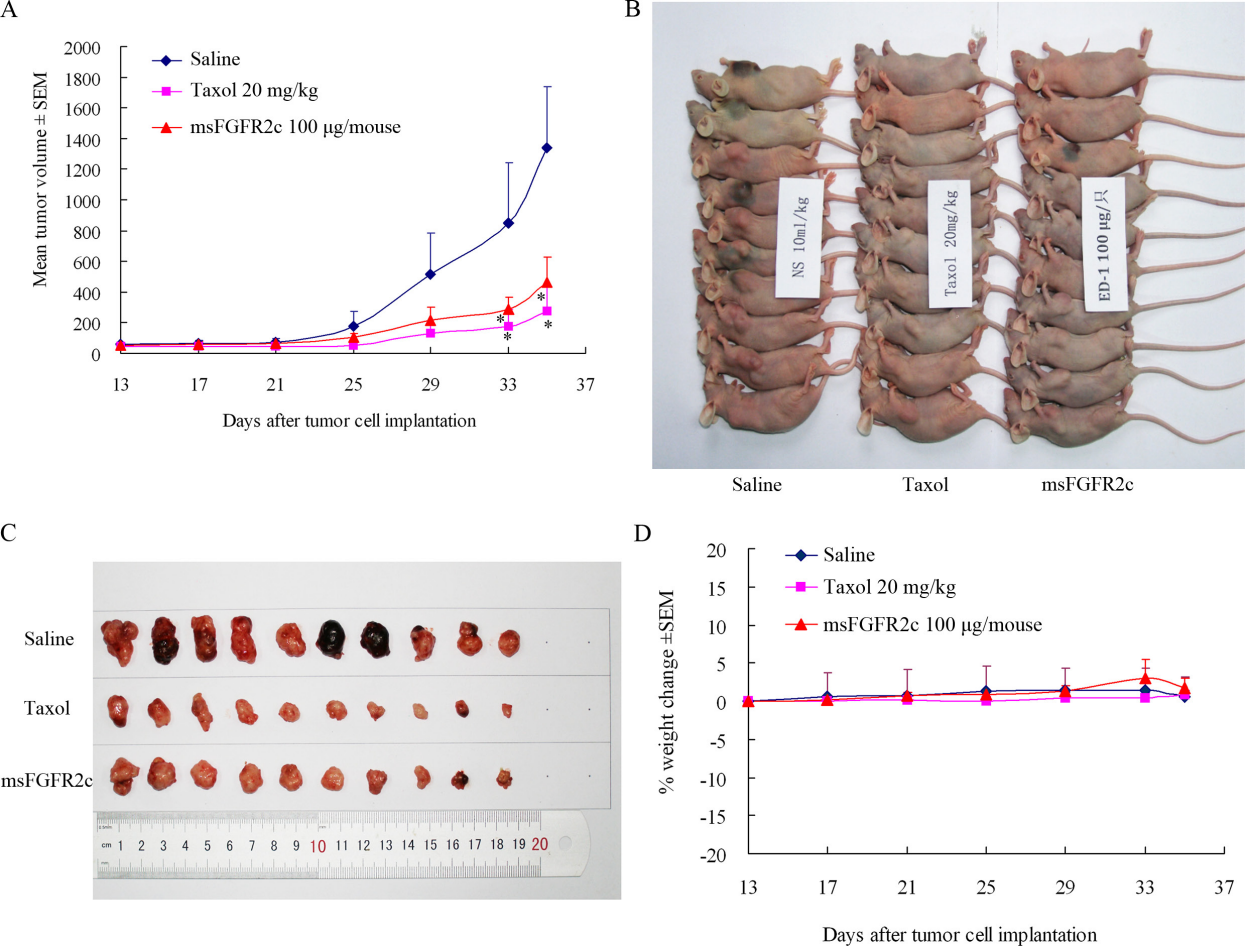
Effects of msFGFR2c on lung cancer growth in lung cancer xenograft model (**A** and **B**) Treatment with msFGFR2c inhibited tumor growth in lung cancer xenograft models. *p < 0.05. The concentrations for saline, taxol and msFGFR2c were 10 ml/kg, 20 mg/kg and 100 μg/mouse, respectively. (**C**) msFGFR2c reduced tumor burden. Pictures of lung cancer xenografts tumor tissue excised from nude mice after euthanization. (**D**) Body weight changes of mouse treated with taxol and msFGFR2c.

The *in vivo* efficacy of msFGFR2c was further determined in a mouse xenograft model. Treatment with msFGFR2c for 22 days led to a significant delay in tumor growth (Figure 11A and 11B). msFGFR2c treatment produced an approximately 70% decrease in tumor weight (Figure 11C) while not affecting normal body weight (Figure 11D).

## DISCUSSION

Some studies have revealed that ectodomain of receptors binds to membrane receptors. Soluble Flt composed of the first six domains of membrane-bound VEGF receptor 1 has been shown to be anti-angiogenesis in several models by acting as a decoy receptor for secreted VEGF and inactivating membrane-bound VEGF receptors 1 (FLT-1) and 2 (KDR) by heterodimerization [11, 12, 27]. Furthermore, VEGF could induce the formation of not only FLT-1 and KDR receptor homodimers but also sFlt and FLT-1/KDR receptor heterodimers allowing the use of the full receptor repertoire [28]. But in these reports, soluble Flt included membrane domain of VEGF receptor. Since receptor dimerization depends on the close of membrane domains, there is a paradox that ectodomain of receptors dimerization also needs the help of membrane domains [29, 30].

It is well known that FGFR activation and signaling are dependent on FGF-induced dimerization, which results in increased kinase activity of FRFR and leading to the ordered phosphorylation of tyrosine residues present on the receptor. These, in turn, recruit and activate a number of signalling pathways required for cell proliferation, migration, and survival. The binding of FGF-2 will lead to conformational changes in D2, D3 and linker domain of FGFR [30], which might confer the ability of FGFR binding to msFGFR2c. It was shown in our study that the proliferation inhibitory effect of msFGFR2c on cancer cells was associated with the expression of different types of FGFRs, implied the binding of msFGFR2c to c subtype of FGFRs. Thus it might not be that msFGFR2c only simply compete with FGF-2 for FGFR in FGF-2-positive cell lines, but rather that ternary complex consisting of FGF-2-FGFR-msFGFR2c might be formed here.

In our study, msFGFR2c, the ectodomain of FGFR2c with S252W mutation, just contained Ig domains of D2 and D3 responsible for the binding of ligands without membrane domain of FGFR2. FGF-2 could induce the formation of heterocomplex between msFGFR2c and membrane-bound full length FGFR2IIIc (Figures 1–3). The results suggested that the formation of such kinds of heterocomplex was not dependent on the membrane domain of FGFRs. The formation of such kind of heterocomplex might be dependent on the S252W mutation since the binding affinity of wsFGFR2c to msFGFR2c was noticeably stronger than that of to wsFGFR2c (Figure 4). Moosa *et al*. previously reported that the S252W mutation would introduce additional interactions between FGFR2 and FGF-2 by the formation of a hydrophobic patch composed of Trp252, Tyr281, and Ile257 of FGFR2 and Phe21 of FGF2, and a side chain hydrogen bond between Tyr281 of FGFR2 and Pro22 of FGF-2 [31]. MD simulations of the present study disclosed two new interactions were formed between msFGFR2c and wsFGFR2c, namely a π-π interaction between the mutated residue Trp252 of msFGFR2c and the residue His254 of wsFGFR2c, and three hydrogen-bond interactions between the residues Arg255 of msFGFR2c and Glu250 of wsFGFR2c (Figure 5). Therefore, the final structure of wsFGFR2c and msFGFR2c heterocomplex could be stabilized by these two interactions, resulting in augmented binding affinity of msFGFR2c to FGFR2c.

Consistently, the antineoplastic effect of sFGFR2c in breast cancer models *in vitro* and *in vivo* demonstrated that msFGFR2c was more potent than wsFGFR2c, as evidenced by cell proliferation, apoptosis, tumor growth and angiogenesis (Figures 6, 7 and 9). The efficacy of msFGFR2c on FGFR2IIIc-positive BT-549 cells was better than FGFR2IIIb-positive/FGFR2IIIc-negative MCF-7 cells and FGFR2IIIb-negative/FGFR2IIIc-negative MDA-MB-231 cells, suggesting that FGFR2IIIc but not FGFR2IIIb was responsible for the binding of msFGFR2c. It should be noted that the inhibitory effect of msFGFR2c could still be observed in FGFR2IIIc-negative MCF-7 cells. That may be due to the binding of msFGFR2c to FGFR1IIIc in MCF-7 cells, since msFGFR2c was also observed to bind to FGFR1IIIc (Figure 1) although the binding amounts were much less than to FGFR2IIIc. Similar results were also observed in FGFR1IIIc-positive NCI-H1299 lung cancer cells. It was demonstrated that msFGFR2c was effective in NCI-H1299 cells *in vitro* and *in vivo* (Figures 10 and 11). These results implied that msFGFR2c might have selectivity in the treatment of cancers expressing c subtype of FGFRs. In addition, high expression level of FGF-2 in cancer cells would be necessary for making msFGFR2c work since FGF-2 is required for the binding of msFGFR2c to the receptor in this study.

In conclusion, we have clarified a novel mechanism for the inhibitory effects of msFGFR2c. msFGFR2c blocked FGF-2/FGFRs signaling pathway by heterocomplexation with c subtype of FGFRs without the involvement of membrane domain of FGFRs. msFGFR2c significantly inhibited cell proliferation, induced apoptosis, suppressed tumor growth and angiogenesis in cancer cells overexpressing FGF-2 and c subtype of FGFRs *in vitro* and *in vivo*. Therefore, msFGFR2c has a potential application for the treatment of FGF-2- and FGFR2c/FGFR1c-positive cancers.

## MATERIALS AND METHODS

### Reagents

Anti-hemagglutinin (HA), anti-phospho-FGFRs (Y653/Y654) were purchased from Cell Signaling Technology. Anti-FGF-2 as well as HRP-goat anti-rabbit conjugate and HRP-goat anti-mouse conjugate were from Santa Cruz. Streptavidin-HRP was purchased from GE Healthcare. Streptavidin, Alexa Fluor 488 conjugate and Dynabeads (R) M-270 Streptavidin were purchased from Invitrogen. IgG-Cy3-goat anti-mouse secondary antibodies were purchased from Chemicon.

### Cell lines and cell culture

Human embryonic kidney cell line 293T, human lung cancer cell line NCI-H1299, human breast cancer cell lines BT-549, MCF-7, and MDA-MB-231 were purchased from the American Type Culture Collection (ATCC, USA). All cells were passaged fewer than 6 months after resuscitation and cultured using the protocol provided by ATCC. Sera and media were purchased from Invitrogen and ATCC.

### Expression, renaturation, and purification of sFGFR2

sFGFR2c (hereafter refer to wsFGFR2c or msFGFR2c) was expressed and purified as described in our previous study [26]. Briefly, cDNA was synthesized from human placenta mRNA by reverse transcription. The DNA region from D2 to D3 of the *FGFR2IIIc* isoform (amino acids 147-366 of hBEK) was amplified from cDNA, and S252W mutation was introduced by splice-overlap PCR method. The fragments of the two genes with or without mutation were cloned into the pET3c vector and expressed in *E. coli*. Bacteria were collected and lysed, and inclusion bodies were isolated. After washing the inclusion bodies twice, protein refolding was performed on a gel filtration column (GE Healthcare, Piscataway, NJ, USA). sFGFR2c with more than 95% purity was harvested through heparin affinity chromatography (GE). In some assays, sFGFR2c was labeled by biotin by DSB-X^™^ Biotin Protein Labeling Kit (Thermo Fisher Scientific, USA) according to the manufacturer's instructions.

### Overexpression of FGFR1IIIb-HA, FGFR1IIIc-HA, FGFR2IIIb-HA, and FGFR2IIIc-HA in HEK 293T cells

cDNA was synthesized from human placenta mRNA by reverse transcription. Full length *FGFR1IIIb*, *FGFR1IIIc*, *FGFR2IIIb*, and *FGFR2IIIc* genes, to which a HA tag was added to the C-terminals, were obtained by PCR amplification. The *Bam*H I and *Hind* III restriction enzyme sites were also added into the ends of the PCR products and the primer sequences are listed in Table 1. The PCR products were cloned into the pCDNA 3.1(−) vector. After sequencing, the plasmids were transfected into HEK 293T cells using Lipofectamine LTX Reagent (Invitrogen).

**Table 1:** Primers used for full length membrane-bound FGFRs

Target	Forward primer (5′ to 3′)	Reverse primer (5′ to 3′)
FGFR1IIIb-HA/	ATATGGATCCGCCGCCACCATG	ATATAAGCTTTCAC ***GCATAGTCAGGAACATCG***
FGFR1IIIc-HA	TGGAGCTGGAAGTGCCTCCTCTT	***TATGGGTAG***CGGCGTTTGAGTCCGCCA
FGFR2IIIb-HA/	ATATGGATCCGCCGCCACCATG	GCGCAAGCTTTCAC***GCATAGTCAGGAACATCGT***
FGFR2IIIc-HA	GTCAGCTGGGGTCGTTTCAT	***ATGGGTA***TGTTTTAACACTGCCGTT

*BamHI* (GGATCC) *and HindIII* (AAGCTT) sites are underlined, and HA-tag sequence is in bold italic.

### Co-immunoprecipitation

HEK 293T cells were transiently transfected with *FGFR1IIIb-HA*, *FGFR1IIIc-HA*, *FGFR2IIIb-HA* or *FGFR2IIIc-HA*, followed by the incubation of 10 μg/ml biotin-labeled msFGFR2c or wsFGFR2c in the presence or absence of FGF-2 for 2 h. The cells were harvested and the lysates were incubated with 50 μl of Dynabeads (R) M-270 Streptavidin at room temperature for 15 min. The beads were washed, harvested and analyzed by immunoblotting with HA and FGF-2 antibodies, respectively.

### Colocalization analysis of sFGFR2c and FGFRs by confocal microscopy

*FGFR2IIIc-HA*, *FGFR1IIIb-HA*, *FGFR1IIIc-HA*, or *FGFR2IIIb-HA* were transiently transfected into HEK 293T cells. The cells were incubated with biotin-labeled msFGFR2c or wsFGFR2c in the presence or absence of FGF-2 at 4°C for 1 h. Cells were fixed, blocked, and incubated with Mouse anti-HA antibodies and cy3-conjugated secondary antibody, then incubated with Streptavidin-Alexa Fluor^®^ 488 and stained with DAPI. Colocalization of sFGFR2c and membrane-bound full length FGFRs was observed by confocal microscope (510 Meta; Carl Zeiss MicroImaging) with excitation wavelengths of 405, 488, and 560 nm.

### Identification of sFGFR2c binding to the surface of FGFR2IIIc-overexpressed HEK 293T cells by flow cytometric analysis

After 24 h of starvation, FGFR2IIIc-overexpressed HEK 293T cells were incubated with biotin-labeled msFGFR2c or wsFGFR2c (2 or 10 μg/ml) in the presence or absence of FGF-2 (20 ng/ml) for 2 h, respetively. Cells were collected and incubated with 4 μg/ml Streptavidin-Alexa Fluor (R) 488 for 30 min. The fluorescence intensity (Alexa Fluor 488) and the percentage of sFGFR2-binding cells were detected using a flow cytometer (FACSAria; BD Biosciences, San Diego, CA, USA) with an excitation wavelength of 488 nm.

### Isothermal titration calorimetry (ITC) measurements

ITC assay were performed with Auto-iTC_200_ (GE, USA). To study the interaction between FGFR2c and msFGFR2c in absence of FGF-2, we used a typical titration consisting of injecting 3 μl aliquots of 150 μM msFGFR2c or 150 μM wsFGFR2c solution into 200 μl aliquots of 20 μM wsFGFR2c solution after every 1 min to ensure that the titration peak returned to the baseline prior to the next injection (wsFGFR2c is the ectodomain of membrane FGFR2IIIc). Aliquots of more concentrated ligand solutions were injected into only the reaction buffer (25 mM HEPES containing 600 mM NaCl and 5% glycerol, pH 7.5) in separate ITC runs to measure the heats of dilution of the ligands. To study the interaction between membrane FGFR2c and msFGFR2c in presence of FGF-2, 20 μM FGFR2IIIc solution was mixed with equal volume *of* 20 μM FGF-2 solution, then the mixed solution was titrated with 150 μM msFGFR2c solution. Control experiments were performed by titrating msFGFR2c into the same buffer to obtain the heats of ligand dilution.

### Molecular dynamics (MD) simulations

The structure of wsFGFR2c, the ectodomain of FGFR2c, was retrieved from Protein Data Bank (PDB code: 1EV2). The chains A, D, E, F were extracted to obtain FGF-2-bound FGFR2c dimer. Missing residues were fixed using the homology module of Molecular Operating Environment 2012 (MOE, Chemical Computing Group Inc, Montreal, Canada). To obtain the initial structure of msFGFR2c, a mutation of serine to tryptophan at position 252 (S252W) on chain E was performed *in silico* from the wsFGFR2c using MOE as well. Then the heterocomplex of msFGFR2c and wsFGFR2c together with their ligand FGF-2 were performed GPU-based MD simulations using PMEMD module in AMBER 12 [32, 33].

For the proteins, the AMBER ff12SB force field was used [34]. The whole system was neutralized by adding sodium/chlorine counter ions and was solvated in TIP3P water molecules with solvent layers of 10 Å between the solute surface and the box edges. The SHAKE algorithm was used to restrict all covalent bonding involving hydrogen atoms with a time step of 2 fs [35, 36]. The Particle-Mesh Ewald (PME) method was applied to treat long-range electrostatic interactions.

Three-step minimization was performed to gradually release the unreasonable contact introduced by system preparation. After minimization, the whole system was heated from 0 to 300 K in 50 ps using Langevin dynamics at a constant volume and then was equilibrated for 500 ps at a constant pressure of 1 atm. Finally, 100 ns periodic boundary dynamic simulations were conducted with an NVT ensemble.

### RNA extraction and real-time quantitative PCR analysis

RNA extraction and real-time quantitative PCR analysis were performed as described in our previous study [26]. Briefly, RNA extraction, reverse transcription, and real-time PCR were performed as previously described [37]. Total RNA was extracted and purified using an RNeasy Kit (Qiagen, Inc., Valencia, CA, USA). Total RNA was reversely transcribed to cDNA using SuperScript II RT-polymerase (Invitrogen Corp., Carlsbad, CA, USA). PCR was performed on the cDNA using primers specific for FGFR1IIIc, FGFR1IIIb, FGFR2IIIc, FGFR2IIIb. The primers used are listed in Table 2.

**Table 2:** Primers used for RT-PCR

Target	Forward primer (5′ to 3′)	Reverse primer (5′ to 3′)
FGFR1IIIc	TCTGGAAGCCCTGGAAGA	GTAGACGATGACCGACCC
FGFR1IIIb	TTGAAGCATTCGGGGATT	CTCTTCCAGGGCTTTTGC
FGFR2IIIc	AGATTGAGGTTCTCTATATTCGGAATG	TTCTCTTCCAGGCGCTGG
FGFR2IIIb	TGCTGGCTCTGTTCAATG	CCAGGCGCTTGCTGTTTT
β-actin	ATTGCCGACAGGATGCAGA	GAGTACTTGCGCTCAGGAGGA

### FGF-2 detection by ELISA

BT-549, MCF-7, and MDA-MB-231 cells were seeded into 100-mm dishes at a density of 2 × 10^6^ per dish and incubated in regular medium overnight then placed in serum-free medium for 24 h. The medium was then removed and stored at −70°C. The FGF-2 level in the medium was determined by using an FGF-2 ELISA assay (Quantikine^™^, R&D, Minneapolis, MN) according to the manufacturer's instructions.

### Bromodeoxyuridine (BrdU) cell proliferation assays

Cells were cultured in 96-well plates (approximately 5000 cells/well) for 24 h. Cells were then starved with phenol red-free DMEM plus 1% dialysed foetal calf serum (A15-107) (PAA laboratories) for 24 h. The experiment included a control group, a FGF-2 group (20 ng/ml), a sFGFR2c group (5 μg/ml), and a FGF-2 plus sFGFR2c group. After 48 h of induction by msFGFR2, 100 μM BrdU was added, and its uptake was measured using colorimetric Cell Proliferation ELISA (Roche). The effect of msFGFR2c on cell viability was assessed as the percentage compared with control, which is arbitrarily assigned 100% viability. The mean value of five wells was calculated, and each experiment was repeated three times.

### DAPI staining

DAPI staining was performed as described previously [38]. Cells were treated with or without 5 μg/ml sFGFR2c for 48 h. In brief, prior to staining, the cells were fixed with 4% paraformaldehyde for 30 min at room temperature, and then washed with PBS. DAPI was added to the fixed cells for 30 min, after which they were examined by fluorescence microscopy. Apoptotic cells were identified by condensation and fragmentation of nuclei.

### Annexin V-FITC/PI dual staining analysis

Annexin V-FITC/PI dual staining analysis was performed to determine cell apoptosis quantitatively. Cells were seeded in 6-well Petri dishes the day prior to the experiment. Cells were treated with or without 5 μg/ml sFGFR2c for 48 h, then harvested, washed twice with ice-cold PBS, and fixed in 70% ethanol at −20°C overnight. Fixed cells were washed once with ice-cold PBS and re-suspended in 1 mL of staining reagent containing 100 mg/mL RNase and 50 mg/mL PI for 25 min in the dark. To assess apoptotic proportion, harvested cells were stained with Annexin-V-FITC/PI (KeyGEN; Nanjing, China) according to the manufacturer's instructions. Fluorescence of PI and Annexin-V-FITC was monitored by low cytometry (BD FACSCalibur; Franklin Lakes, CA, USA) at 630 nm and 525 nm, respectively.

### Effect of msFGFR2c on FGFRs activation in BT-549, MCF-7, and MDA-MB-231 cells

BT-549, MCF-7, and MDA-MB-231 cells were incubated with sFGFR2c or FGF-2 at 37°C for 30 min. The experiment included a control group, a FGF-2 group (20 ng/ml), a sFGFR2c group (320 ng/ml), and a FGF-2 plus sFGFR2c group. Cells were fixed, blocked, and incubated with anti-phospho-FGFRs (Y653/Y654) antibody and cy3-conjugated secondary antibody and stained with DAPI and were observed under a confocal microscope (510 Meta; Carl Zeiss MicroImaging) with excitation wavelengths of 405 and 560 nm.

### Tumor growth in the chick chorioallantoic membrane (CAM) cancer implant model

Fertilized chicken eggs were maintained under constant humidity at 37°C. A small window was made in the shell on day 4 of chick embryo development under aseptic conditions. The window was resealed with adhesive tape and eggs were returned to the incubator until day 8 of chick embryo development. When studying the effect of msFGFR2c *in vivo*, on day 8, BT549, MCF-7, MDA-MB-231 cell suspensions (2 × 10^7^) were inoculated onto each CAM, and eggs were resealed and returned to the incubator (*n* = 8 chicken embryos per cell line). On day 10, inoculated cells were treated with msFGFR2c (at 4 μg/egg/d) in CAM. Tumors were harvested 3 days later until day 13. Quantification of the vascular network of CAM was assessed by counting the number of vessels converging toward the implant under a stereomicroscope and the experiment was repeated three times.

### Lung cancer xenograft experiments

Fifteen nu/nu athymic BALB/c male mice (4-6 weeks old) were obtained from the Experimental Animal Center of Sun Yat-sen University (Guangzhou, China). All animal experiments were performed in compliance with Institutional Animal Care and Use Committee guidelines. Tumors were established by flank injection of 5 × 10^6^ cells suspended with reconstituted basement membrane (BD Biosciences, Bedford, MA) at a ratio of 2:1 (volume). When tumors reached 100 mm^3^, mice were randomly divided into three groups: control (*n* = 8), msFGFR2c (*n* = 8) and taxol (*n* = 8). Animals were received alternate day intravenous injection of msFGFR2c (100 μg) or an equal volume of saline for 22 days. Tumor volumes were calculated using the formula: 1/2 × larger diameter × (smaller diameter)^2^. Mice were sacrificed and tumors were excised and weighed.

### Statistical analysis

Statistical analysis was performed using the Statistical Package for Social Sciences 13.0 (SPSS). Data are presented as mean ± s.e.m. Student's *t* test (two-tailed) was used to compare two groups for independent samples assuming equal variances among all experimental data sets. Statistical significance was assumed if *p* < 0.05.
